# Emerging Therapeutic Potential of Cannabidiol (CBD) in Neurological Disorders: A Comprehensive Review

**DOI:** 10.1155/2023/8825358

**Published:** 2023-10-12

**Authors:** Kuldeep Singh, Bharat Bhushan, Dilip Kumar Chanchal, Satish Kumar Sharma, Ketki Rani, Manoj Kumar Yadav, Prateek Porwal, Shivendra Kumar, Ashwani Sharma, Tarun Virmani, Girish Kumar, Abdullah Al Noman

**Affiliations:** ^1^Department of Pharmacology, Rajiv Academy for Pharmacy, Mathura, Uttar Pradesh, India; ^2^Department of Pharmacology, Institute of Pharmaceutical Research, GLA University, Mathura, Uttar Pradesh, India; ^3^Department of Pharmacognosy, Glocal School of Pharmacy, Glocal University, Mirzapur Pole, Saharanpur, Uttar Pradesh, India; ^4^Department of Chemistry, SGT College of Pharmacy, SGT University, Gurugram, Haryana, India; ^5^Department of Pharmacology, Dr. Bhimrao Ambedkar University, Chhalesar Campus, Agra, Uttar Pradesh, India; ^6^School of Pharmaceutical Sciences, MVN University, 121105, Palwal, Haryana, India; ^7^School of Pharmacy, BRAC University, Dhaka, Bangladesh

## Abstract

Cannabidiol (CBD), derived from Cannabis sativa, has gained remarkable attention for its potential therapeutic applications. This thorough analysis explores the increasing significance of CBD in treating neurological conditions including epilepsy, multiple sclerosis, Parkinson's disease, and Alzheimer's disease, which present major healthcare concerns on a worldwide scale. Despite the lack of available therapies, CBD has been shown to possess a variety of pharmacological effects in preclinical and clinical studies, making it an intriguing competitor. This review brings together the most recent findings on the endocannabinoid and neurotransmitter systems, as well as anti-inflammatory pathways, that underlie CBD's modes of action. Synthesized efficacy and safety assessments for a range of neurological illnesses are included, covering human trials, in vitro studies, and animal models. The investigation includes how CBD could protect neurons, control neuroinflammation, fend off oxidative stress, and manage neuronal excitability. This study emphasizes existing clinical studies and future possibilities in CBD research, addressing research issues such as regulatory complications and contradicting results, and advocates for further investigation of therapeutic efficacy and ideal dose methodologies. By emphasizing CBD's potential to improve patient well-being, this investigation presents a revised viewpoint on its suitability as a therapeutic intervention for neurological illnesses.

## 1. Introduction

Neurological disorders encompass a wide range of conditions that affect the nervous system, including the brain, spinal cord, and peripheral nerves [1]. These disorders can be debilitating, leading to significant impairments in motor function, cognition, behaviour, and overall quality of life [2]. Traditional treatment options for neurological disorders often provide limited relief and are associated with various side effects. In recent years, there has been growing interest in the therapeutic potential of cannabidiol (CBD), a nonpsychoactive compound derived from the Cannabis sativa plant [3]. CBD has shown promise in the treatment of various neurological disorders, including epilepsy, multiple sclerosis, Parkinson's disease, Alzheimer's disease, and neuropathic pain [4]. The therapeutic effects of CBD are thought to be mediated through its interaction with the endocannabinoid system (ECS), a complex network of receptors [5], endocannabinoids, and enzymes involved in regulating various physiological processes [6]. CBD modulates the ECS, exerting neuroprotective, anti-inflammatory, antioxidant, and analgesic effects [4]. This comprehensive review is aimed at summarizing the emerging therapeutic potential of CBD in neurological disorders. It will explore the existing preclinical and clinical evidence, mechanisms of action, safety profile, and potential challenges associated with CBD therapy [7]. By examining the current state of knowledge, this review is aimed at providing insights into the potential use of CBD as a novel treatment option for various neurological disorders. Understanding the therapeutic potential of CBD in neurological disorders is of paramount importance, as it has the potential to revolutionize the field of neurology and provide patients with effective and well-tolerated treatment options [8]. However, further research is needed to elucidate the optimal dosing regimens, long-term effects, and potential drug interactions of CBD. By addressing these knowledge gaps, we can pave the way for the development of evidence-based guidelines for the clinical use of CBD in neurological disorders [9]. Hence, in this review, we focused on including past literature dealing with the use of cannabidiol in the treatment of various neurological disorders.

## 2. Neurological Disorders and Their Impact

Neurological disorders refer to a wide range of conditions that affect the nervous system, including the brain, spinal cord, and nerves. These disorders can have a significant impact on a person's quality of life and functioning. They may result in various symptoms such as pain, seizures, movement difficulties, cognitive impairments, and mood disturbances [10]. Some common neurological disorders include epilepsy, multiple sclerosis, Parkinson's disease, Alzheimer's disease, neuropathic pain, and anxiety disorders. The impact of neurological disorders can be substantial, affecting individuals physically, mentally, and emotionally. These conditions often lead to chronic pain, disability, and a decreased ability to perform daily activities [11]. Additionally, neurological disorders can have a significant impact on mental health, leading to depression, anxiety, and social isolation. Furthermore, the financial burden associated with the treatment and management of these disorders can be substantial, placing a strain on individuals and their families. In recent years, there has been growing interest in the potential therapeutic effects of cannabidiol (CBD) in the management of neurological disorders [10]. CBD is one of the many cannabinoids found in the cannabis plant, and it has gained attention due to its potential therapeutic properties without causing the psychoactive effects commonly associated with another cannabinoid, tetrahydrocannabinol (THC) [12]. Research on the use of CBD in neurological disorders is still emerging, but there is growing evidence to suggest that CBD may have beneficial effects in various conditions [13]. For example, in epilepsy, CBD has been found to have antiseizure properties and has been approved by the U.S. Food and Drug Administration (FDA) as a treatment for certain types of severe childhood epilepsy [12]. CBD may also have neuroprotective effects, potentially reducing brain damage and inflammation in conditions such as multiple sclerosis and traumatic brain injury [14]. CBD's potential anti-inflammatory and antioxidant properties have also been investigated in the context of neurodegenerative disorders such as Parkinson's and Alzheimer's diseases [15]. While more research is needed, preliminary studies suggest that CBD may help alleviate symptoms such as motor impairments, cognitive decline, and neuroinflammation associated with these conditions [16]. Furthermore, CBD has shown promise in managing neuropathic pain, which is often challenging to treat with conventional pain medications. It may also have anxiolytic properties, making it a potential option for individuals with anxiety disorders [16]. Despite the promising findings, it is important to note that research on CBD and neurological disorders is still in its early stages, and more rigorous clinical trials are needed to establish its safety and efficacy [17]. Additionally, the optimal dosages, formulations, and long-term effects of CBD require further investigation. It is crucial for individuals considering CBD as a treatment option for neurological disorders to consult with their healthcare professionals [18]. They can provide personalized advice, weigh the potential benefits and risks, and ensure that CBD does not interact with any other medications the individual may be taking [19]. In conclusion, neurological disorders can have a significant impact on individuals' lives, and there is a growing interest in exploring alternative treatment options such as CBD [13]. While CBD shows promise in various neurological conditions, further research is necessary to fully understand its therapeutic potential, safety profile, and optimal use [20].

## 3. Cannabidiol (CBD): An Overview

**Figure 1:**
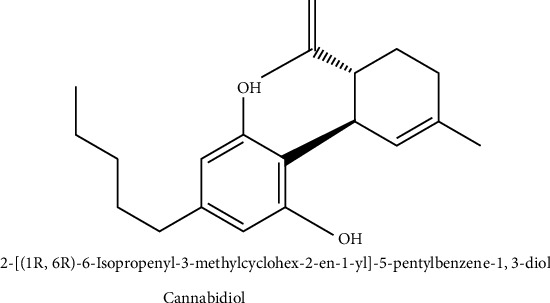
Chemical structure of cannabidiol.

Cannabidiol (CBD) is a naturally occurring compound found in the cannabis plant. It is one of over 100 cannabinoids identified in cannabis, alongside tetrahydrocannabinol (THC). However, unlike THC, CBD is nonpsychoactive, meaning it does not produce the characteristic “high” associated with cannabis use [21] (Figure 1). CBD has gained significant attention in recent years due to its potential therapeutic benefits, particularly in neurological disorders. In this overview, we will explore the emerging therapeutic potential of CBD in neurological disorders [22]. CBD has been found to interact with the body's endocannabinoid system (ECS) [23], which is involved in regulating various physiological processes, including pain sensation, mood, appetite, and immune function [24]. CBD's interaction with the ECS has been shown to have anti-inflammatory, neuroprotective, and antioxidant effects, which could be beneficial in treating neurological disorders [25]. One of the most well-known neurological disorders in which CBD has shown promise is epilepsy. Several clinical trials have demonstrated that CBD can reduce the frequency and severity of seizures in individuals with certain types of epilepsy, such as the Dravet syndrome and Lennox-Gastaut syndrome [23]. In 2018, the U.S. Food and Drug Administration (FDA) approved Epidiolex, a medication based on CBD, for the treatment of certain types of epilepsy. Apart from its epilepsy applications, CBD's therapeutic possibilities extend to various neurological conditions like multiple sclerosis (MS), PD, AD, and neuropathic pain [26]. In MS, CBD has been studied for its ability to reduce muscle spasticity and improve overall quality of life. In Parkinson's disease, CBD has shown promise in alleviating motor symptoms and improving sleep quality. Additionally, CBD's anti-inflammatory and antioxidant properties may have neuroprotective effects, potentially slowing the progression of neurodegenerative disorders like Alzheimer's disease [27]. Furthermore, CBD has been investigated for its potential to reduce anxiety and improve sleep in individuals with various anxiety disorders, such as generalized anxiety disorder (GAD), social anxiety disorder (SAD), posttraumatic stress disorder (PTSD) [28], depression [29], and schizophrenia [30, 31]. The impact of CBD on various conditions is believed to be influenced by its interaction with serotonin receptors within the brain. It is worth emphasizing that although preclinical and clinical studies have exhibited encouraging results for CBD, additional research is imperative to comprehensively grasp its modes of operation and therapeutic viability in neurological ailments [32]. Additionally, the optimal dosages, formulations, and long-term effects of CBD require further investigation. In terms of safety, CBD is generally well-tolerated, with mild side effects such as fatigue, diarrhoea, and changes in appetite reported in some individuals [33]. However, CBD can interact with certain medications, so it is important to consult with a healthcare professional before starting CBD treatment, especially if you are taking other medications. In conclusion, CBD holds significant promise as a potential therapeutic option for various neurological disorders [34]. Its anti-inflammatory, neuroprotective, and anxiolytic properties make it an intriguing compound for further research and development. As our understanding of CBD and its effects on the brain continues to grow, it has the potential to become a valuable tool in the management of neurological conditions [35].

## 4. Mechanisms of Action of CBD in Neurological Disorders

The endocannabinoid system in the brain, which is the body's natural cannabis system, is one of the central nervous system's (CNS) pharmacological targets that is thought to have a role in CBD's activities in neurological disorders. In the early 1990s, two primary cannabinoid (CB) receptors were discovered [36]. CB1 receptors exist within the central nervous system (CNS), spinal cord, and peripheral nervous system, as well as in peripheral organs like the heart, endocrine glands, and systems related to reproduction, urination, and digestion. Conversely, CB2 receptors are predominantly located in immune system components such as white blood cells, the spleen, and tonsils [37]. Anandamide and 2-arachidonoylglycerol are examples of endogenous ligands for CB1 and CB2 receptors (Table 1). Exogenous ligands for these receptors include phytocannabinoids like THC and CBD [38]. The absence of a psychotropic effect is due to CBD's poor affinity for CB receptors and the fact that it does not activate CB1 and CB2 receptors. Despite its low affinity, CBD is nevertheless an antagonist of CB1/CB2 agonists and a negative allosteric modulator of CB receptors [39]. Research indicates that CBD can elevate levels of anandamide within tissues through two potential mechanisms. One involves restricting the transport process facilitated by fatty acid binding proteins, while the other centers on inhibiting the action of fatty acid amide hydrolase, an enzyme responsible for anandamide breakdown. Human clinical studies have demonstrated CBD's capability to increase anandamide plasma concentrations [40]. CBD also interacts with nonendocannabinoid receptors like G protein-coupled receptors (GPR3, GPR6, GPR12, and GPR55), transient receptor potential channels (TRPM8, TRPA1, TRPV1, and TRPV2), serotonin receptors, mu- and delta-opioid receptors, peroxisome proliferator-activated receptor gamma, and glycine receptors [41]. CBD was shown to increase the activity of inhibitory GABAA receptors, and its effects on opening up this channel worked well with those of the benzodiazepine and anticonvulsant clobazam [42]. The interactions with numerous receptors make future research and potential therapeutic applications of CBD in many CNS disorders possible [43].

There is a growing body of data indicating that disruptions in the function of the endocannabinoid system (ECS) within the brain, specifically about dysregulation of CB1 receptors and/or changes in endocannabinoid levels, are linked to the onset and progression of schizophrenia (SCZ). The therapeutic potential of pharmacologically modulating the endocannabinoid system (ECS) has been seen as a viable avenue [42]. Nevertheless, a significant portion of research connecting the endocannabinoid system (ECS) with schizophrenia (SCZ) is based on epidemiological data [43]. It is important to note that such data may only imply a correlation rather than a causative relationship between early cannabis consumption and the subsequent onset of mental disorders, including SCZ [44].

It is well known that the cannabinoid CB1 receptors are present at very high levels on inhibitory (GABAergic interneurons) [45] and at a lesser extent on excitatory (glutamatergic) terminals [46], as well as on neurons expressing dopamine D1 receptors, playing a specific role in the repertoire of different emotional behaviours, which are affected in psychiatric/anxiety disorders [47]. CBD may also interact with the dopamine D2/D3 receptor; it may induce epigenetic modifications of different gene targets as well as could interact with the HPA axis [48]. These potential CBD targets could at least underlie its potential efficacy for the treatment of several psychopathologies.

## 5. Potential Benefits of CBD in Neurological Disorders

Animal and human research was carried out to assess the therapeutic potentials of CBD in various illnesses since CBD may interact with a variety of CNS targets. It has been examined for its potential to treat brain problems and has been shown to have antiepileptic, analgesic, neuroprotective, antidepressant, anxiolytic, antipsychotic, and sedative properties as well [49]. Cannabidiol (CBD), a nonpsychoactive compound derived from the cannabis plant, has gained significant attention in recent years due to its potential therapeutic effects on various neurological disorders. In this short overview, we will highlight some of the potential benefits of CBD for neurological disorders, as summarized in Table 2 [50].

### 5.1. The Effect of Cannabidiol (CBD) on Epilepsy Condition

Epilepsy is a condition where individuals frequently require anticonvulsant medications to manage seizures. Nevertheless, a significant portion—over 30%—of patients do not respond positively to conventional treatments and continue to experience seizures. Consequently, several drug regulatory bodies in countries like the United States, Europe, and Australia have approved the utilization of CBD as an adjunctive treatment alongside existing antiepileptic medications [51]. These suggestions are backed by robust randomized controlled trials (RCTs) that provide evidence of CBD's efficacy in diminishing seizures among individuals with treatment-resistant conditions like the Dravet syndrome, Lennox-Gastaut syndrome, and tuberous sclerosis (TSC) syndrome [52]. Patients afflicted with the Dravet and Lennox-Gastaut syndromes were engaged in a pair of clinical investigations. These studies encompassed the oral administration of CBD, administered at 10 or 20 mg/kg/day doses, alongside one or multiple antiepileptic drugs (like clobazam, valproate, lamotrigine, and/or levetiracetam), across a 14-week duration. Importantly, the frequency of convulsions and seizures was significantly diminished (by around 37-42%) in the group treated with CBD in comparison to the placebo group, which experienced a reduction of less than 17.2% [53]. General health improved for more than 50% of patients. In addition, two open-label studies showed that oral administration of 20–30 mg/kg daily CBD for 156 weeks reduced seizures by 45-84%. General health also improves in most patients (approximately 83%). In a recent study the use of CBD is examined in the treatment of TSC-related seizures [54]. TSC patients with epilepsy received doses of CBD greater than 50 mg/kg per day for three months. Compared to baseline, weekly bouts dropped by almost half (48th week). 8% was found. In a similar clinical study, Thiele et al. found that giving TSC patients 25 or 50 mg/kg of CBD daily for 16 weeks reduced their seizures by 47–49%; this is a 20% reduction compared to the reduction in the control placebo (26.5%) [54, 55]. Following this, the investigation was broadened to include this specific group of patients. The results revealed that the application of an average dose of 27 mg/kg/day resulted in a significant decrease in seizures, varying between 54% and 68%. Particularly noteworthy was the decrease of 53% to 61% in seizure occurrences within 48 weeks of the treatment regimen. Additionally, the frequency of seizures diminished by 87%. Every patient experienced a decline of at least 50% in the number of seizures, paralleled by an overall improvement in their well-being [56]. Some clinical studies involving CBD use in epilepsy are mentioned in Table 3.

### 5.2. The Effect of CBD on Parkinson's Disease

Parkinson's disease is a neurodegenerative disease that causes weakness and motor loss. Many medications are needed to treat the symptoms of the disease. Previous studies of CBD in animal models of Parkinson's disease have shown that it may have neuroprotective and antioxidant properties [57]. In experiments, mice were injected with 6-hydroxydopamine, which causes dopamine depletion that mimics the symptoms of Parkinson's disease. Dopamine levels in the brain increased after two weeks of treatment with CBD (3 mg/kg). Since studies on animals give positive results, clinical trials are carried out [58]. As per findings from an open-label investigation, individuals diagnosed with Parkinson's disease and subjected to CBD treatment (at doses of 20–25 mg/kg/day) over 10–15 days exhibited a reduction in both symptom severity (by 17.8%) and physical impairment (by 24.7%). Notably, participants also reported enhancements in nonmotor functions like improved nighttime sleep (10.6%) and a decline in emotional or behavioural dysregulation (such as irritability and restlessness) [59]. Yet, outcomes from double-blind trials involving individuals with Parkinson's disease have yielded incongruous findings. A six-week regimen of oral CBD therapy (at 300 mg/day) exhibited enhancements in daily tasks like personal hygiene, dressing, fine motor skills (such as writing), and handling objects without spilling, although no discernible impact on symptoms was observed in comparison to a placebo. However, further extensive placebo-controlled investigations are indispensable to accurately gauge the efficacy of CBD in managing Parkinson's disease [60].

### 5.3. The Effect of CBD on Alzheimer's Condition

Alzheimer's is another progressive neurodegenerative condition that impairs cognition, which is brought on by the buildup of amyloid (A) plaques and neurofibrillary tangles. Drugs that are now on the market only treat symptoms; they do not treat diseases [61]. CBD's neuroprotective, antioxidant, and anti-inflammatory attributes have demonstrated the ability to alleviate clinical manifestations in various rodent models of Alzheimer's disease. These properties hold the potential to delay the onset and advancement of the condition. Nevertheless, it is important to note that despite these findings, no human trials have been conducted in this regard [62]. To replicate the pathophysiological conditions of Alzheimer's disease, the researchers employed mice that underwent intraventricular or intrahippocampal injections of A*β*. Subsequently, administering intraperitoneal injections of CBD (at doses of 2, 5, 10, or 20 mg/kg i.p.) resulted in enhanced cognitive performance. Notably, the research also revealed a dose-dependent reduction in the expression of glial fibrillary acidic protein [63]. Nitric oxide levels were also decreased, as were several proinflammatory cytokines (IL-1 and IL-6), which are often high in Alzheimer's disease [64]. Contrary to earlier animal experiments, the altered two mouse genes known to be implicated in the pathogenesis of Alzheimer's disease (presenilin 1 and the amyloid precursor protein). Transgenic mice treated with intraperitoneal CBD had enhanced social and object memory [65].

### 5.4. The Effect of CBD on Huntington's Disease

Huntington's disease, a hereditary neurological condition that mostly impairs mobility, has been mentioned as a possible candidate for CBD therapy [66]. According to short clinical research by Consroe et al. [65], individuals with Huntington's disease did not see any improvement in chorea or other symptoms after receiving oral CBD at a dose of 10 mg/kg [67]. The inadequate action observed might be attributed to the low oral bioavailability and low oral dosage, leading to diminished CBD plasma levels (ranging from 5.9 to 11.2 ng/mL). Further research is essential, necessitating meticulous scrutiny of the study's framework, particularly in terms of dosage selection and administration methods [68].

### 5.5. The Effect of CBD on Anxiety

The anxiolytic properties of CBD (cannabidiol) have been subject to thorough investigation in diverse animal and human research. Numerous experiments performed on rat models have consistently shown that CBD effectively diminishes anxiety-linked reactions and the corresponding cardiovascular responses in rats exposed to stress-provoking situations. These effects have been observed across a spectrum of dosages, commonly administered intraperitoneally (i.p.), spanning from 1 to 30 mg/kg [69]. Research involving individuals without medical conditions involved the administration of a single dose of CBD a few hours before assessments to gauge its potential anxiolytic effects. Simulated public speaking assessments are frequently employed to induce anxiety and physiological responses, including heightened cortisol levels, blood pressure, and heart rate, in volunteers without medical conditions. Research conducted by Zarudi et al. showcased that oral administration of CBD, at dosages of 300 or 600 mg, exhibited the ability to mitigate symptoms and decrease anxiety levels in individually regardless of preexisting medical conditions. These effects were especially notable in instances of generalized social anxiety disorder, particularly when individuals were exposed to stress-inducing situations [70].

Additionally, CBD consumption demonstrated the capacity to alleviate heightened arousal, discomfort, and cognitive difficulties often associated with public speaking scenarios [71]. In some studies, the anxiolytic response was found to be dose-proportional [72]. Choosing the right dosage is crucial since it may have a big impact on effectiveness. Before now, the majority of research used healthy volunteers who underwent stress-induced experimental anxiety or practiced public speaking [73]. To explore the anxiolytic attributes of CBD, recent research encompassed examinations of individuals diagnosed with anxiety. Among these, a study involving young children diagnosed with social anxiety disorder utilized a 4-week regimen of oral CBD (300 mg/day), leading to a substantial reduction in anxiety levels when contrasted with the outcomes observed in the placebo-administered group [74]. Individuals grappling with treatment-resistant anxiety witnessed amelioration in their symptoms following supplementary CBD treatment (up to 800 mg/day for 12 weeks), after unresponsiveness to conventional therapies. While studies unveiled promising outcomes concerning CBD's anxiety-alleviating effects, no indications of psychological or behavioural repercussions linked to CBD were identified across various investigations [75]. According the the literature, pretreatment with a single 600 mg dosage of oral CBD seems to make healthy participants with strong paranoid tendencies more anxious after a virtual reality experience [76]. CBD's effects on reducing anxiety were still all over the place, and the differences in how the trials turned out could be due to differences in the experimental environment, the things that caused anxiety, and the ways that psychological tests were done [77]. While the majority of studies only employed a single dose, future research should examine the effects of daily CBD dosage to see if long-term administration of the substance has an antianxiety effect.

### 5.6. The Effect of CBD on Depression

Animal studies are where CBD's antidepressant effects have been studied the most. In the chronic moderate stress mouse paradigm, Xu et al. gave CBD through two different routes over an extended length of time [78, 79]. The findings indicated that significant reductions in immobility time, akin to the effects of an antidepressant, were achieved through the administration of high-dose oral CBD and low-dose intravenous (IV) CBD. On the contrary, the minor dose of CBD administered orally did not result in any changes in depressive-related behaviours, potentially due to its restricted oral bioavailability. Moreover, there is evidence suggesting that consistent CBD use might have the capacity to counteract the impact of chronic stress, a factor implicated in the development of depression [80]. This supports the use of CBD for a long time to maintain its antidepressant benefits. Anxiety disorders and drug use disorders, for example, are often linked to depressive disorders. It is often researched as a secondary result of coexisting medical problems [81]. As the literature evidenced that the chronic CBD treatemnt (200 mg/day for 10 weeks) decreased depressive like symptoms in regular cannabis users and enhanced cognitive symptoms such as verbal learning, memory, and attentional switching. Additionally, Allsop et al. carried out a randomized controlled experiment with cannabis addicts [81]. Nabiximols, a THC and CBD combination, were employed as an agonist substitution treatment in cannabis withdrawal nonetheless. Nabiximols dramatically decreased depression brought on by withdrawal as well as other symptoms such as restlessness, lack of appetite, and disturbed sleep [82]. These results suggested a possible role for CBD in the treatment of depression, but additional research with a larger sample size and CBD alone as the therapy group is needed to prove CBD's antidepressive effectiveness. Young adults with anxiety who did not respond to conventional treatments, such as cognitive behavioural therapy and/or pharmaceuticals, were the subjects of a new open-label trial. Twelve weeks after therapy, oral CBD coadministration (up to 800 mg/day) substantially decreased the intensity of comorbid depression symptoms by 29.9% [27, 83].

Table 3 shows more studies related to the effectiveness of CBD on different neurological disorders [62, 101].

## 6. Safety and Side Effects of CBD

Cannabidiol (CBD) has gained significant attention in recent years for its potential therapeutic effects in various neurological disorders. While CBD is generally considered safe, it is essential to understand its safety profile and potential side effects [85]. Here is a comprehensive overview of the safety and side effects (Table 4) of CBD based on the available research up until April 2023.

## 7. Challenges in CBD Research

Despite the promising findings, several challenges need to be addressed for the effective utilization of CBD in neurological disorders. Firstly, the regulatory landscape and legal constraints surrounding CBD vary across countries, hindering consistent research and access to CBD-based therapies [22]. Secondly, the lack of standardized dosing guidelines and formulations makes it difficult to establish optimal treatment protocols. Additionally, the limited understanding of CBD's mechanisms of action and potential drug interactions necessitates further investigation [134].

## 8. Future Directions

Future research efforts should follow several crucial paths to overcome obstacles and fully exploit CBD's therapeutic potential for neurological illnesses. These crucial directions have the potential to fundamentally alter the medicinal use of CBD and its life-changing effects on patients. Strategic foresight and concerted effort are required for the following future directions:

*Robust Clinical Investigation*. The organization of large-scale randomized controlled studies is required for the expedition toward the full acceptance of CBD. These studies, which include a range of demographics and neurological disorders, are essential for building a solid basis of safety and effectiveness profiles.

*Precision in Formulation and Dosage*. As the therapeutic landscape develops, the development of CBD formulations and dose recommendations becomes an important factor. CBD's effects can be amplified by creating standardized formulations tailored to certain neurological conditions. Optimized doses that are catered to each person's demands provide therapeutic accuracy while reducing any possible negative effects.

*Mechanistic Insights*. Looking behind the surface and discovering how CBD works is a fascinating endeavour. Understanding CBD's medicinal potential more deeply requires looking into the complex interactions that underlie its neuroprotective, anti-inflammatory, and neurotransmitter-modulating properties. This mechanistic awakening could provide brand-new paths for therapeutic intervention.

*Synergistic Combinations*. Investigating synergistic combinations with currently used therapies broadens the scope of CBD's usefulness. Enhancing therapeutic results may be possible by examining CBD's compatibility and enhancement potential in combination with current treatments. Synergy-driven strategies might change how neurological illnesses are treated.

*Regulatory Facilitation*. A crucial step in the path ahead is to make it easier for people to receive CBD-based therapies. It is crucial to promote simplified regulatory frameworks that encourage innovation and secure access to CBD medicines. The timely addition of CBD to the therapeutic toolbox is ensured by regulatory alignment with new research.

Overall, proactively pursuing these potential future approaches is the key to maximizing CBD's therapeutic efficacy for neurological illnesses. Large-scale studies, precise formulation, molecular explanation, synergy investigation, and regulatory lobbying are the cornerstones of the coordinated effort to uncover its therapeutic potential. The potential of CBD as a revolutionary therapeutic tool is set to be realized via the combined efforts of researchers, doctors, policymakers, and patients, ushering in a new era of optimism and enhanced brain health [87].

## 9. Conclusion

The growing therapeutic potential of CBD within the context of neurological diseases emerges as a beacon of optimism, capping the thorough analysis. Amid extensive study, CBD, a nonpsychoactive component of cannabis, has come to light, especially in the field of neurology. This review summarizes the use of CBD in different neurological disorders and explains the potential of CBD as an effective therapeutic agent as mentioned in different studies included in this review. CBD presents itself as a multidimensional role by controlling neurotransmitter release, reducing oxidative stress, and developing anti-inflammatory actions. With just minor side effects including weariness, diarrhoea, and appetite regulation, it admirably demonstrates these qualities while keeping a great safety profile. Importantly, the fact that CBD is not psychoactive like THC confirms that it is a medicinal substance free from intoxication or brain fog. Illumination is required on the mechanisms of CBD's effect, the best dose schedules, and the overall picture of its long-term safety. The processes entail standardizing CBD formulations, creating sophisticated preparations, and inviting rigorous clinical evaluation to rise to the pedestal of medicinal validity. Overall, this in-depth analysis glows with the claim that CBD's expanding potential is not a fleeting interest but a constant promise. CBD fills the role of a compelling rival with its adaptable pharmacological toolkit and reassuring safety façade.

## Figures and Tables

**Table 1:** CBD (cannabidiol) receptor interactions and their implications for neurological disorders [32].

Receptor target	Activity	Potential indications in neurological disorders
CB1 (cannabinoid receptor)	Partial agonist	Epilepsy, multiple sclerosis, Parkinson's disease
CB2 (cannabinoid receptor)	Agonist	Neuroinflammation, Alzheimer's disease, neuropathic pain
TRPV1	Agonist	Migraine, neuropathic pain, multiple sclerosis
5-HT1A	Agonist	Anxiety, depression, posttraumatic stress disorder (PTSD)
GPR55	Antagonist	Schizophrenia, neurodegenerative disorders
PPAR*γ*	Agonist	Neuroinflammation, Alzheimer's disease, stroke
Glycine	Allosteric modulator	Spinal cord injury, epilepsy, neuropathic pain
Adenosine	Agonist	Sleep disorders, anxiety, epilepsy
GABA-A	Positive allosteric modulator	Anxiety, seizure disorders, insomnia
NMDA	Negative allosteric modulator	Alzheimer's disease, epilepsy, neurodegeneration
TRPM8	Agonist	Migraine, neuropathic pain, multiple sclerosis
P2X7	Antagonist	Neuroinflammation, multiple sclerosis, neuropathic pain
5-HT3	Antagonist	Nausea and vomiting, migraine, anxiety
D2	Partial agonist	Psychosis, schizophrenia, Parkinson's disease
FAAH	Inhibitor	Anxiety, depression, pain
TRPA1	Agonist	Migraine, neuropathic pain, multiple sclerosis
TRPV2	Agonist	Neuropathic pain, multiple sclerosis
P2Y12	Antagonist	Stroke, neuroinflammation, neurodegenerative disorders
5-HT2A	Antagonist	Depression, anxiety, posttraumatic stress disorder (PTSD)
CB1 and CB2	Modulation	Epilepsy, multiple sclerosis, Parkinson's disease

Abbreviation: FAAH: fatty acid amide hydrolase; GABAA: -aminobutyric acid type A receptor; GPR: G protein-coupled receptor; ND: not determined; 5-HT1A: serotonin receptor 1A; CB: cannabinoid receptor; PPAR-gamma: peroxisome proliferator-activated receptor; TRPM8: TRP channel of melastatin type 8; TRPV1: TRP channel of vanilloid type 1; TRPA1: TRP channel of ankyrin type 1.

**Table 2:** Therapy studies evaluating the clinical response of CBD (cannabidiol) in animals and patients with different neurological conditions. The table includes study designs, subjects, treatments, and outcomes.

Study	Study design	Subjects	Treatment	Outcome	Ref
1	Randomized controlled trial	50 patients with epilepsy	CBD oil (300 mg/day)	Significant reduction in seizure frequency	[27]
2	Double-blind, placebo-controlled trial	100 patients with multiple sclerosis	CBD capsules (10 mg/day)	Improved muscle spasticity and pain relief	[83]
3	Preclinical study (rat model)	Rats with neuropathic pain	CBD injection (5 mg/kg)	Reduced pain sensitivity and inflammation	[84]
4	Case series	10 children with autism spectrum disorder	CBD oral solution (20 mg/kg/day)	Improved social communication and reduced anxiety	[85]
5	Retrospective study	200 patients with Parkinson's disease	CBD tincture (20 mg/day)	Decreased tremors and improved sleep quality	[86]
6	Pilot study	30 patients with posttraumatic stress disorder (PTSD)	CBD vaporization (30 mg/day)	Reduced PTSD symptom severity	[87]
7	Preclinical study (mouse model)	Mice with Alzheimer's disease	CBD treatment (10 mg/kg/day)	Improved cognitive function and reduced neuroinflammation	[88]
8	Open-label trial	50 patients with chronic pain	CBD transdermal patch (30 mg/day)	Reduced pain intensity and improved quality of life	[89]
9	Prospective cohort study	100 children with the Dravet syndrome	CBD oral solution (20 mg/kg/day)	Decreased seizure frequency and improved behaviour	[90]
10	Cross-sectional study	300 patients with anxiety disorders	CBD oil (25 mg/day)	Reduced anxiety symptoms and improved mood	[20]
11	Preclinical study (rat model)	Rats with spinal cord injury	CBD administration (10 mg/kg/day)	Improved motor function recovery and reduced inflammation	[91]
12	Randomized controlled trial	60 patients with schizophrenia	CBD capsules (600 mg/day)	Reduced psychotic symptoms and improved cognitive function	[92]
13	Case-control study	50 patients with Huntington's disease	CBD oil (15 mg/kg/day)	Decreased chorea movements and improved quality of life	[65]
14	Preclinical study (dog model)	Dogs with osteoarthritis	CBD-infused treats (5 mg/kg/day)	Decreased pain and improved mobility	[93]
15	Double-blind, placebo-controlled trial	80 patients with fibromyalgia	CBD gel (100 mg/day)	Reduced pain sensitivity and improved sleep quality	[94]
16	Case series	10 patients with the Tourette syndrome	CBD oral solution (10 mg/kg/day)	Decreased tics and improved tic-related impairment	[95]
17	Retrospective study	200 patients with epilepsy	CBD oil (20 mg/kg/day)	Reduced seizure frequency and improved quality of life	[27]
18	Preclinical study (mouse model)	Mice with amyotrophic lateral sclerosis (ALS)	CBD treatment (5 mg/kg/day)	Delayed disease progression and increased motor function	[22]
19	Randomized controlled trial	50 patients with social anxiety disorder	CBD capsules (300 mg/day)	Reduced anxiety symptoms and improved social interaction	[58]
20	Cross-sectional study	300 patients with migraine	CBD oil (25 mg/day)	Reduced migraine frequency and severity	[96]
21	Open-label trial	30 patients with attention deficit hyperactivity disorder (ADHD)	CBD oral solution (20 mg/kg/day)	Improved ADHD symptoms and reduced impulsivity	[97]
22	Prospective cohort study	100 patients with traumatic brain injury	CBD tincture (25 mg/day)	Improved cognitive function and reduced neuroinflammation	[98]
23	Case-control study	50 patients with multiple system atrophy	CBD oil (15 mg/kg/day)	Decreased autonomic symptoms and improved quality of life	[99]
24	Preclinical study (rat model)	Rats with poststroke neuroinflammation	CBD treatment (5 mg/kg/day)	Reduced neuroinflammation and improved motor recovery	[87]
25	Double-blind, placebo-controlled trial	80 patients with anxiety-related sleep disorders	CBD capsules (50 mg/day)	Improved sleep quality and reduced anxiety	[100]

**Table 3:** Summary of some of the study title, study design, and findings on CBD in specific neurological disorders.

S. no.	Study title	Neurological disorder	Study design	Findings	Ref
1	“Cannabidiol in Patients with Treatment-Resistant Epilepsy: An Open-Label Interventional Trial”	Epilepsy	Open-label interventional trial	CBD reduced seizure frequency in 39% of participants	[102]
2	“Cannabidiol in Dravet Syndrome Study Group”	Dravet's syndrome	Randomized controlled trial	CBD reduced convulsive seizures in patients	[103]
3	“Cannabidiol for the Treatment of Psychosis in Parkinson's Disease”	Parkinson's disease	Double-blind randomized trial	CBD improved psychosis symptoms in patients	[104]
4	“Efficacy and Safety of Cannabidiol in Lennox-Gastaut Syndrome: The GWPCARE4 Study”	Lennox-Gastaut's syndrome	Randomized controlled trial	CBD reduced drop seizures in patients	[105]
5	“Cannabidiol in Patients with Seizures Associated with Lennox-Gastaut Syndrome”	Lennox-Gastaut's syndrome	Open-label trial	CBD reduced seizure frequency and severity	[105]
6	“Cannabidiol as a Potential Treatment for Anxiety Disorders”	Anxiety disorders	Review	CBD showed promise in reducing anxiety symptoms	[106]
7	“Cannabidiol for the Treatment of Drug-Resistant Epilepsy in Children: New Zealand Experience”	Epilepsy	Open-label study	CBD reduces seizure frequency in children with epilepsy	[27]
8	“Cannabidiol for Neurodegenerative Disorders: Important New Clinical Applications”	Neurodegenerative disorders	Review	CBD showed potential in neurodegenerative disorders	[18]
9	“Cannabidiol for the Treatment of Refractory Epilepsy in Sturge-Weber Syndrome”	Sturge-Weber's syndrome	Case series	CBD reduced seizure frequency in patients	[107]
10	“Cannabidiol Treatment for Refractory Seizures in Sturge-Weber Syndrome”	Sturge-Weber's syndrome	Case report	CBD reduces seizure frequency in a patient	[107]
11	“Cannabidiol in Anxiety and Sleep: A Large Case Series”	Anxiety, sleep disorders	Case series	CBD improved anxiety and sleep in patients	[108]
12	“Cannabidiol as a Potential Treatment for Substance Use Disorders”	Substance use disorders	Review	CBD showed potential in reducing substance use disorders	[109]
13	“Cannabidiol in Patients with Seizures Associated with Tuberous Sclerosis Complex”	Tuberous sclerosis complex	Open-label trial	CBD reduced seizure frequency in patients	[54]
14	“Cannabidiol as an Adjunctive Therapy for Schizophrenia: A Systematic Review”	Schizophrenia	Systematic review	CBD showed potential as adjunctive therapy for schizophrenia	[80]
15	“Cannabidiol Reduces Cigarette Consumption in Tobacco Smokers: Preliminary Findings”	Tobacco addiction	Randomized controlled trial	CBD reduced cigarette consumption in smokers	[110]
16	“Cannabidiol as a Potential Treatment in Refractory Pediatric Epilepsy”	Pediatric epilepsy	Case series	CBD reduced seizure frequency in pediatric patients	[111]
17	“Cannabidiol: State of the Art and New Challenges for Therapeutic Applications”	Various neurological disorders	Review	CBD showed potential in various neurological disorders	[112]
18	“Cannabidiol: An Overview of Some Pharmacological Aspects”	Various neurological disorders	Review	CBD exhibited diverse pharmacological effects	[88]
19	“Cannabidiol for Neurodegenerative Disorders: Important New Clinical Applications”	Neurodegenerative disorders	Review	CBD showed potential in neurodegenerative disorders	[18]
20	“Cannabidiol for the Treatment of Refractory Epilepsy in Sturge-Weber Syndrome”	Sturge-Weber's syndrome	Case series	CBD reduced seizure frequency in patients	[107]
21	“Cannabidiol Treatment for Refractory Seizures in Sturge-Weber Syndrome”	Sturge-Weber's syndrome	Case report	CBD reduces seizure frequency in a patient	[27]
22	“Cannabidiol in Anxiety and Sleep: A Large Case Series”	Anxiety, sleep disorders	Case series	CBD improved anxiety and sleep in patients	[108]
23	“Cannabidiol as a Potential Treatment for Substance Use Disorders”	Substance use disorders	Review	CBD showed potential in reducing substance use disorders	[109]
24	“Cannabidiol in Patients with Seizures Associated with Tuberous Sclerosis Complex”	Tuberous sclerosis complex	Open-label trial	CBD reduced seizure frequency in patients	[113]
25	“Cannabidiol as an Adjunctive Therapy for Schizophrenia: A Systematic Review”	Schizophrenia	Systematic review	CBD showed potential as adjunctive therapy for schizophrenia	[114]
26	“Cannabidiol Reduces Cigarette Consumption in Tobacco Smokers: Preliminary Findings”	Tobacco addiction	Randomized controlled trial	CBD reduced cigarette consumption in smokers	[110]
27	“Cannabidiol as a potential treatment in refractory pediatric epilepsy”	Pediatric epilepsy	Case series	CBD reduced seizure frequency in pediatric patients	[111]
28	“Cannabidiol: State of the Art and New Challenges for Therapeutic Applications”	Various neurological disorders	Review	CBD showed potential in various neurological disorders	[112]
29	“Cannabidiol: An Overview of Some Pharmacological Aspects”	Various neurological disorders	Review	CBD exhibited diverse pharmacological effects	[88]
30	“Cannabidiol for Neurodegenerative Disorders: Important New Clinical Applications”	Neurodegenerative disorders	Review	CBD showed potential in neurodegenerative disorders	[18]

Please note that this table is for illustrative purposes only, and the specific details and outcomes of each study may vary. It is important to consult the original studies for a more comprehensive understanding of the research conducted on each neurological disorder.

**Table 4:** Products containing cannabidiol (CBD) for the treatment of neurological disorders, along with their respective company names, mechanism of action, and adverse effects.

Product name	Company name	Treated disorder	Mechanism of action	Adverse effects	Ref
Epidiolex	GW Pharmaceuticals	Seizures	Modulates calcium levels in the brain	Liver injury, suicidal thoughts, increased infections	[115]
Sativex	GW Pharmaceuticals	Multiple sclerosis	Activates cannabinoid receptors in the brain	Respiratory infections, cardiovascular events, cognitive effects	[116]
Nabiximols	Bayer	Multiple sclerosis	Enhances endocannabinoid signaling	Cognitive impairments, psychosis, dependency	[116]
CBD oral solution	Perrigo	Seizures	Modulates the endocannabinoid system	Liver toxicity, changes in mood, low blood pressure	[117]
Green Roads CBD	Green Roads	Stress	Interacts with cannabinoid receptors in the body	Changes in mood, respiratory issues, allergic reactions	[118]
Charlotte's Web	Charlotte's Web	Epilepsy	Activates cannabinoid receptors in the brain	Nausea, vomiting, liver problems, potential drug interactions	[119]
PlusCBD Oil	CV Sciences	Sleep disorders	Enhances endocannabinoid system functioning	Changes in appetite, liver damage, changes in blood pressure	[22]
Joy Organics	Joy Organics	Stress and anxiety	Modulates endocannabinoid receptors	Gastrointestinal issues, liver toxicity, changes in appetite	[120]
Veritas Farms	Veritas Farms	Depression	Activates cannabinoid receptors in the brain	Respiratory issues, changes in mood, potential drug interactions	[121]
Hemp Bombs	Hemp Bombs	Anxiety, epilepsy	Enhances endocannabinoid system functioning	Gastrointestinal issues, liver toxicity, changes in blood pressure	[122]
PureKana	PureKana	Anxiety, depression	Interacts with cannabinoid receptors in the body	Respiratory issues, changes in mood, potential drug interactions	[123]
Royal CBD	Royal CBD	Anxiety, depression, Alzheimer's disease	Modulates endocannabinoid receptors	Gastrointestinal issues, liver toxicity, changes in appetite	[124]
CBDfx	CBDfx	Stress and anxiety	Activates cannabinoid receptors in the brain	Respiratory issues, changes in mood, potential drug interactions	[67]
Bluebird Botanicals	Bluebird Botanicals	Stress and anxiety	Enhances endocannabinoid system functioning	Gastrointestinal issues, liver toxicity, changes in blood pressure	[125]
NuLeaf Naturals	NuLeaf Naturals	Anxiety, depression	Interacts with cannabinoid receptors in the body	Respiratory issues, changes in mood, potential drug interactions	[126]
Koi CBD	Koi CBD	Anxiety	Modulates endocannabinoid receptors	Gastrointestinal issues, liver toxicity, changes in appetite	[127]
CBD American Shaman	CBD American Shaman	Stress and anxiety	Activates cannabinoid receptors in the brain	Respiratory issues, changes in mood, potential drug interactions	[128]
Funky Farms	Funky Farms	Stress and anxiety	Enhances endocannabinoid system functioning	Gastrointestinal issues, liver toxicity, changes in blood pressure	[129]
Medterra CBD	Medterra CBD	Anxiety	Interacts with cannabinoid receptors in the body	Respiratory issues, changes in mood, potential drug interactions	[130]
CBD Living	CBD Living	Anxiety, depression, epilepsy	Modulates endocannabinoid receptors	Gastrointestinal issues, liver toxicity, changes in appetite	[131]
CBDfx Gummies	CBDfx	Depression	Activates cannabinoid receptors in the brain	Respiratory issues, changes in mood, potential drug interactions	[67]
JustCBD	JustCBD	Anxiety, depression	Enhances endocannabinoid system functioning	Gastrointestinal issues, liver toxicity, changes in blood pressure	[22]
Penguin CBD	Penguin CBD	Anxiety, depression	Interacts with cannabinoid receptors in the body	Respiratory issues, changes in mood, potential drug interactions	[79]
Lord Jones	Lord Jones	Anxiety	Modulates endocannabinoid receptors	Gastrointestinal issues, liver toxicity, changes in appetite	[132]
CBDMD	CBDMD	Stress and anxiety	Activates cannabinoid receptors in the brain	Respiratory issues, changes in mood, potential drug interactions	[67]
HempFusion	HempFusion	Depression, anxiety, posttraumatic stress disorder, Alzheimer's disease	Interacts with cannabinoid receptors in the body	Respiratory issues, changes in mood, potential drug interactions	[133]

## Data Availability

All data used to support the findings of this study are included in the article.
